# Reading strategies in Stargardt's disease with foveal sparing

**DOI:** 10.1186/1756-0500-3-15

**Published:** 2010-01-22

**Authors:** Mira Goldschmidt, Anouk Déruaz, Erika N Lorincz, Andrew R Whatham, Christophe Mermoud, Avinoam B Safran

**Affiliations:** 1 Ophthalmology Clinic, Department of Clinical Neurosciences, Geneva University Hospitals, Alcide-Jentzer 22, 1211 Geneva 14, Switzerland; 2 Perception and Eye Movement Laboratory, Department of Neurology and Department of Clinical Research, University of Bern, Inselspital, Switzerland; 3 Institute for Eye Research, University of New South Wales, Sydney, Australia

## Abstract

**Background:**

Subjects with a ring scotoma can use two retinal loci, a foveal and a peripheral, for reading. Our aim was to investigate the relative use of both retinal loci as a function of the spared foveal area size and the spatial resolution at both retinal loci.

**Findings:**

Two patients with Stargardt's disease and ring scotomas read through a scanning laser ophthalmoscope a series of letters and words at various character sizes. The number of fixations made using each retinal locus was quantified. The relative use of each retinal locus depended on character size of the stimulus. Both patients used exclusively the eccentric retinal locus to read words of large character sizes. At small character sizes, the central retinal locus was predominantly used. For reading letters or words, once foveal fixation was used, patients did not shift back to the eccentric retinal locus. When spatial resolution allowed deciphering at both the eccentric and the central areas, patients consistently fixated with the eccentric retinal locus.

**Conclusions:**

Spatial resolution at the eccentric locus appears as a determinant factor to select the retinal area for reading. Reading strategies in patients with Stargardt's disease and a ring scotoma demonstrate a pattern of coordination of both eccentric and central retinal loci, reflecting a high degree of adaptation.

## Background

Central scotomas are frequently encountered in low vision populations and induce an impediment to efficient reading. Common causes of central scotomas include age-related macular degeneration (AMD) and Stargardt's disease. Patients with central scotoma use an extrafoveal locus for fixation in place of the fovea [1]. Such a locus is commonly referred to as preferred retinal locus (PRL) [2].

Some patients affected by macular degeneration develop a ring scotoma. Such a condition is of particular interest with regard to its functional implications. As previously reported [3-5], subjects with a ring scotoma fixate small stimuli with the central locus, but wider stimuli with the eccentric locus. The alternation between central and eccentric fixations was found in 8 out of 40 eyes of patients with Stargart's disease [6], and it was suggested that this fixation pattern was a phase in the transition from foveal to eccentric fixation [7].

In the present study we investigated reading strategies in patients with Stargardt's disease and a ring scotoma. Our purpose was to assess the relative use of two PRLs, a foveal and a peripheral, as a function of the spared foveal area size and of the spatial resolution at both PRLs. The results showed that reading strategies strongly depended on the spatial resolution at the eccentric retinal locus.

## Methods

### Patients

Two female patients (CC and CN) with bilateral ring scotomas following Stargardt's disease were studied. They were aged 28 (CC) and 36 years (CN). The time since onset of the macular disease was 5 and 2 years respectively. None of the patients had received either low vision rehabilitation or optical magnifiers before data collection. Patients gave their informed consent to the testing procedures that were in accordance with the Declaration of Helsinki.

### Experimental set-up and testing procedures

**Figure 1:**
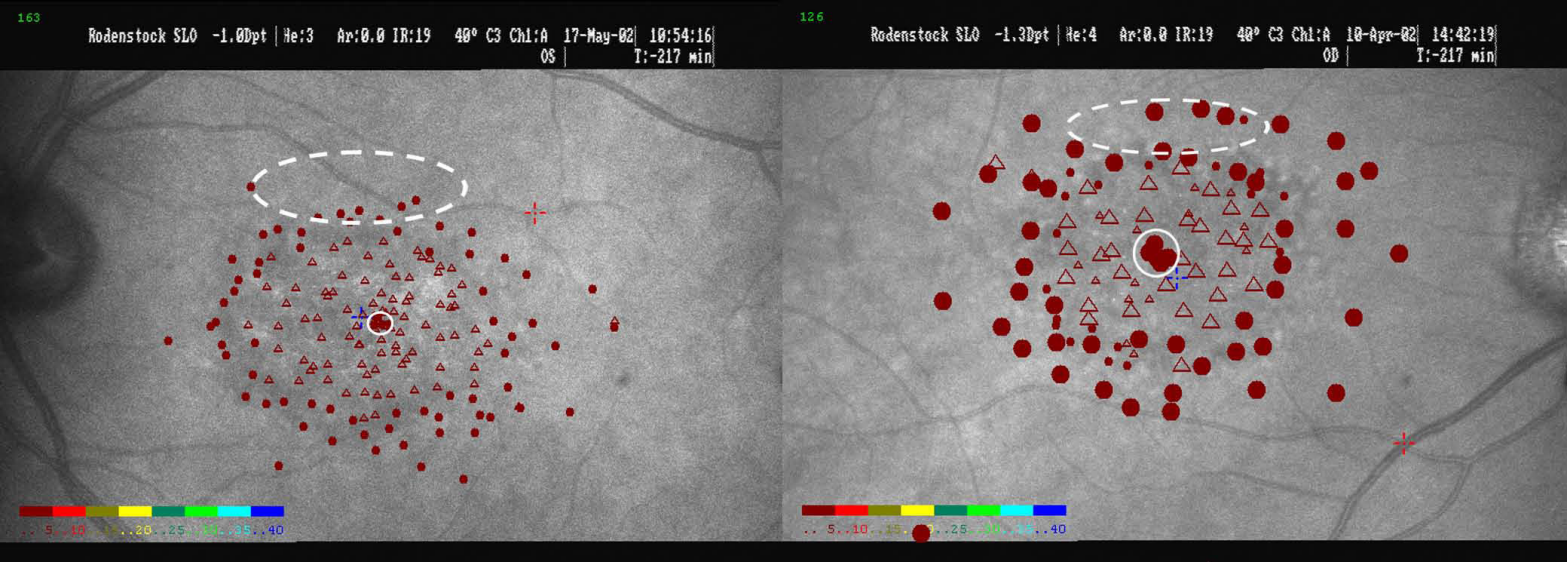
**SLO pictures of the functional lesion and locations of the PRLs**. The SLO images show the scotoma and the spared central area for patients CC (left) and CN (right). The retinal lesion was delineated by microperimetry using Goldmann III target size (for CN: III and IV target sizes). Plain circles and open triangles represent respectively perceived and non-perceived stimuli. The colour scale represents the stimulus intensity in dB, from the highest intensity (red) to the lowest (blue). The central PRL was further assessed with a laser beam projected onto a Maddox cross. The location of the central PRL is denoted by a plain white circle. The location of the eccentric PRL is represented by a dashed white ellipse, as we did not define precisely its extent, the later depending at least in this study, of the stimulus size.

Investigations were conducted on the eye showing the best acuity, according to a method previously described elsewhere [8]. Briefly, the examination procedure consisted in three sessions. In the first session, visual acuity was determined for both eyes with ETDRS charts. To delineate the scotoma, a microperimetry was performed within the central 15 degrees of the retina using the Rodenstock scanning laser ophthalmoscope (SLO), by means of Goldman III targets of 200 cd/m^2 ^in intensity (Figure 1). In the second session, patients were asked to read aloud through the SLO single letters and isolated words of eight different character sizes, decreasing by 0.1 logMAR steps, from 1.5 to 0.8 logMAR. A total of 32 isolated letters and words of 2, 5, and 10 letters were presented at random locations and in a random order within the field of view of the instrument. During the reading task, images of the ocular fundus and the superimposed word stimuli were recorded onto videotapes at a frequency of 25 frames per second. In the third session, the spatial resolution at the central and the eccentric PRLs was measured using the Visumetry program of the SLO.

### Data analysis

**Figure 2:**
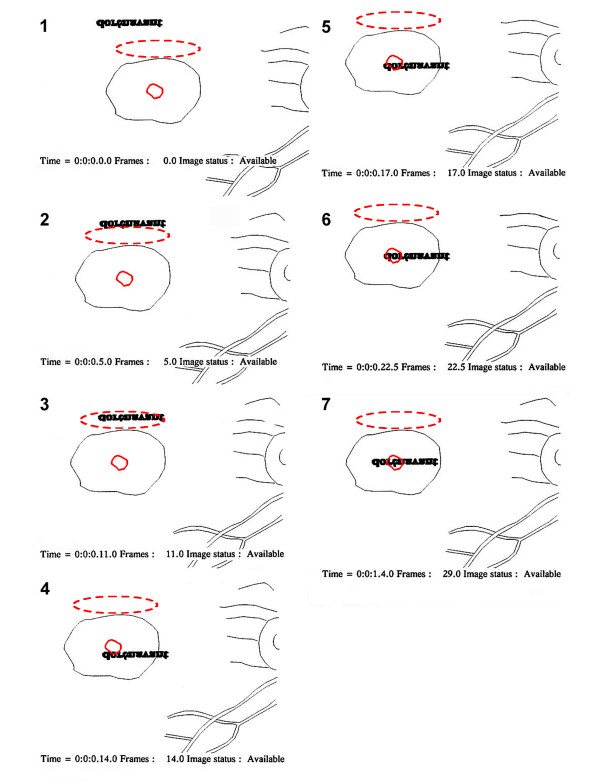
**Example of fixations performed with the eccentric and the central PRLs to read "dorénavant"**. Successive fixations performed with the eccentric (dashed red) and the central PRLs (plain red) are shown for patient CN (right eye) while reading the word "dorénavant" (bold characters). Character size was 0.9 logMAR, i.e., 0.6° of visual angle on average. The time (hour:minute:second.frame) and the starting frame number of the picture ("Image status: Available") are shown beneath each image. The word appeared on an eccentric position (image 1) and was progressively (image 2) brought adjacent to the lower border of the retinal lesion where is located the eccentric PRL (image 3). A further saccade positioned the word adjacent to the central PRL (image 4), and into it (image 5). Finally several horizontal saccades along the word completed deciphering the word (images 6, 7). Note that this figure represents retinal pictures as seen through the SLO.

The locations of the central and the eccentric PRLs were determined after the testing, with a computer program developed in our laboratory [9]. Images of the ocular fundus and the superimposed word stimuli were digitised and converted into schematic representations. Eye movements were automatically extracted by comparing the shift of the eye fundus position relative to the word as it is read. Sequences of eye movements during reading were represented as "cartoons" of the ocular fundus and the superimposed word (Figure 2). The PRLs are defined as being the retinal areas consistently used to fixate and read letters and words.

After locating the PRLs, the number of fixations was manually quantified, through frame-by-frame playback of the video recording. A fixation was defined as a stable retinal position of at least 3 successive frames, i.e., 120 ms. Reading strategies were assessed by the proportions of fixations performed with either the eccentric or the central PRL. The proportions of fixations were calculated for each subject and each letter or word. To determine the relative use of both PRLs as a function of character size and words length, the coefficient  was computed from the number of fixations made with each PRL.

## Results

### Patients' clinical characteristics

Both patients used two distinct PRLs for reading, one central and the other eccentric. The central PRL was at or close to the fovea. The eccentric PRL was always located at the same area on the retina, above the macular lesion, i.e., below the scotoma in the visual field. The central and the eccentric PRLs were separated by approximately 5 degrees. Patients' clinical characteristics are shown in Table 1. As the spared central area size was 0.75° and 1.3° in diameter for patients CC and CN respectively, it could fit letters up to 1.1 logMAR (CC) and 1.3 logMAR (CN). At the smallest character size (0.8 logMAR), only 1.32 (CC) and 2.28 (CN) letters on average could potentially fit into the central area.

**Table 1:** Patients' clinical characteristics

Patients	Eye	Visual Acuity (logMAR)	Scotoma size	Spatial resolution at the eccentric PRL (logMAR)	Central PRL size	Spatial resolution at the central PRL (logMAR)
CN	OD	0.26	10°	1.0	1.3°	Better than 0.8*

CC	OS	0.2	10°	1.2	0.75°	Better than 0.8*

*: 0.8 logMAR is the spatial resolution limit of the SLO visumetry program.

Spatial resolution at the eccentric PRL, as determined by the visumetry test, was 1.2 logMAR and 1.0 logMAR for patients CC and CN respectively. As could be expected from the EDTRS tests, the spatial resolution at the central PRL was better than 0.8 logMAR for both patients, which is the SLO's program spatial resolution limit.

### Reading strategies

**Figure 3:**
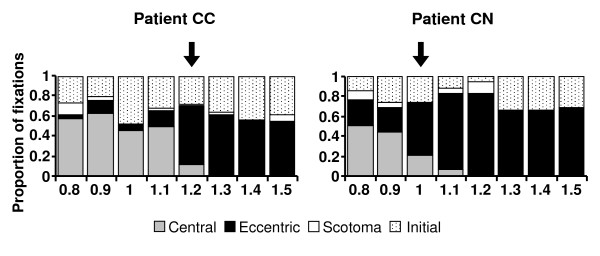
**Proportions of fixations as a function of character sizes**. The proportions of fixations made previous to fixating the word with the PRL ("Initial": dotted pattern) and those performed while reading with the eccentric PRL ("Eccentric": black), the central PRL ("Central": grey) and the intervening "fixations" in the scotoma ("Scotoma": white) are represented as a function of character sizes (logMAR) for patients CN and CC. The arrow shows the spatial resolution threshold at the eccentric PRL. Note the shift from an exclusive use of the eccentric PRL to read the largest character sizes to increasing use of the central PRL as character size decreased.

**Figure 4:**
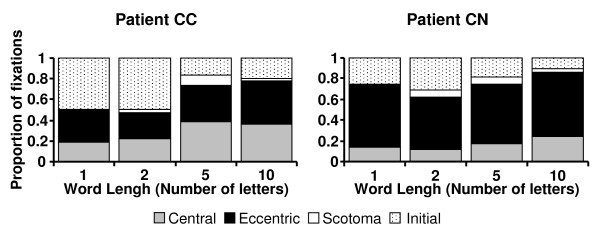
**Proportions of fixations as a function of word length**. The proportions of fixations made previous to fixating the word with the PRL ("Initial": dotted pattern) and those performed while reading isolated letters and words with the eccentric PRL ("Eccentric": black), the central PRL ("Central": grey) and the intervening "fixations" in the scotoma ("Scotoma": white) are represented as a function of the number of letters in the word (word length) for patients CN and CC. Note that the proportions of eccentric and central fixations are similar across word length.

Both patients used similar reading strategies. At the appearance of the word, fixations were made with one or several eccentric loci of the retina, before the word be fixated with the PRL (Figure 3 and Figure 4). The following fixations were performed using the eccentric or the central PRL. Once starting to read with the central PRL, they did not shift back to the eccentric PRL. Occasionally, stimuli were transiently shifted into the scotoma area during reading.

For both patients the relative use of the central PRL and the eccentric PRL strongly depended on the character size (Figure 3). The analysis performed on the PC coefficient revealed that the proportion of fixations between the eccentric and the central PRLs varied significantly across the 8 character sizes (Kruskal-Wallis test, CC: χ^2 ^= 24.73, df = 7, p = 0.001; CN: χ^2 ^= 22.44, df = 7, p = 0.002), although the total number of fixations did not differ significantly between character sizes (non parametric Kruskal-Wallis test, CC: χ^2 ^= 3.44, df = 7, p = 0.841; CN: χ^2 ^= 7.30, df = 7, p = 0.398). Patients used exclusively the eccentric PRL to read words of large character sizes (CC: 1.3 logMar; CN: 1.2 logMar). They mainly used eccentric fixations until 1.2 logMAR (CC) and 1 logMAR (CN). At the smallest character size they employed predominantly the central PRL.

In contrast to character size, word's length did not influence the relative use of each PRL (Figure 4). The proportion of eccentric or central fixations was similar across word length for each patient (Kruskal-Wallis test, CC: χ^2 ^= 0.82, df = 3, p = 0.85; CN: χ^2 ^= 0.06, df = 3, p = 0.996). As expected, patients made more fixations for reading longer words (Kruskal-Wallis test, CC: χ^2 ^= 19.69, df = 3, p < 0.0005; CN: χ^2 ^= 8.60, df = 3, p = 0.035).

## Discussion

In both patients with Stargardt's disease and a ring scotoma reading strategies involved the combined use of both the eccentric PRL and the central PRL. The relative use of each PRL depended on character size. For reading words with large character sizes, patients used exclusively the eccentric PRL. For smaller character sizes, patients combined benefits of the eccentric PRL, i.e. a wider visual span and presumably a better spatial word localisation, with those of the preserved foveal zone, i.e. a higher spatial resolution.

The use of multiple PRLs has been attributed to several factors. Whittaker [10] noticed that multiple PRLs were more likely to occur with large central scotoma above 20°, while Crossland [11] reported higher occurrence in patients with recent vision loss (<12 weeks).

The selection and the location of the PRLs also depend on the task requirements, including visual acuity, visual span, stimulus and background luminance, correspondence in binocular vision, attention load [12-16]. Alternation between multiple PRLs with complementary functional advantages might improve performance of the visual task.

Our findings emphasised the importance of the spatial resolution at the eccentric PRL as one of the determinant factor in selecting a fixation locus for reading. Interestingly, when characters could be deciphered in both PRLs, patients consistently used the eccentric locus. Although patients had a better spatial resolution at the central than at the eccentric PRL, they did not shift fixation to the central PRL as soon as one letter could fit within it, but only when character size was below the resolution of the eccentric PRL.

In contrast to character size, word length did not appear to influence the relative use of the central or the eccentric PRL. Although the central area could accommodate more than 1 or 2 letters, patients selected the eccentric PRL as long as its spatial resolution enabled reading.

Another interesting finding was that once foveal fixation was used, patients did not shift back to the eccentric PRL. This phenomenon can be attributed to the fact that using the eccentric PRL implies the development of new oculomotor skills, involving shift of the oculomotor reference from the fovea to a non foveal locus [17]. Subsequently, eye movements need to be recoded in another coordinate system as soon as changes in PRLs occur, which is a constraining task, requiring a major plastic reorganisation in the oculomotor control.

## Conclusions

Reading strategies in patients with Stargardt's disease and a ring scotoma demonstrate a pattern of coordination of both eccentric and central PRLs. The spatial resolution at the eccentric PRL was a determinant factor in selecting a fixation locus. When spatial resolution allowed deciphering in both eccentric and central areas, patients consistently fixated with the eccentric PRL. More studies with greater patients' samples are needed to confirm these preliminary results.

## Competing interests

The authors declare that they have no competing interests.

## Authors' contributions

MG participated in the design of the study, the acquisition, the analysis and the interpretation of the data and the drafting of the manuscript. AD participated in the design of the study, the acquisition, the analysis and the interpretation of the data, and the drafting of the manuscript. ENL participated in the analysis and the interpretation of the data, and the drafting of the manuscript. ARW participated in the design of the study and the analysis of the data. CM designed the computer software necessary to analyse the data. ABS participated in the design of the study, in the interpretation of the data and in drafting the manuscript. All authors read and approved the final manuscript.
